# The regulatory challenges of innovative customized combination products

**DOI:** 10.3389/fmed.2022.821094

**Published:** 2022-07-22

**Authors:** Mariana E. Reis, Ana Bettencourt, Helena M. Ribeiro

**Affiliations:** Pharmacy, Pharmacology and Health Technologies, Research Institute for Medicines (iMed.ULisboa), Faculdade de Farmácia, Universidade de Lisboa, Lisboa, Portugal

**Keywords:** combination products, drug-device, regulatory aspects, principal mode of action, custom-made devices, 3D-printed scaffold

## Abstract

**Background/aims:**

Combination products are therapeutic and/or diagnostic products that can combine drugs and medical devices and which increasing complexity has raised new regulatory framework challenges. To reach the market, a combination product must be classified based on the principal mode of action (PMOA). However, research and technological progress has been leading to the development of novel combination products with no clearly defined PMOA, emphasizing the lack of a systematization process, thus challenging the correct classification of these products. To illustrate the regulatory challenge, two case studies are discussed: innovative combination products with PMOA that can change due to an external stimulus, specifically custom-made 3D-printed scaffolds with incorporated medicinal substances.

**Methods:**

Data was collected through computational search engines, regulatory agencies and equally relevant associations. The analysis of the data resulted on this state-of-the-art review, a description of the decision-making process by the regulatory authorities, and case studies analysis that culminated in the proposal of a decision-tree scheme.

**Findings:**

Current regulations do not fully address complex combination products namely personalized 3D-printed scaffolds. Two merged regulatory approaches are suggested along with the schematization of the rational assisted by a decision-tree tool.

**Conclusion:**

Combination products have become increasingly sophisticated, which has furthered the need to develop multidisciplinary collaborations within the health sector to adapt to these innovative healthcare solutions as well as with regulators to overcome the challenges posed for their classification.

## Introduction

In the early 2000s, the industry began to realize that the future of therapeutic interventions was not limited to narrow categories of medical devices, drugs, or biological products. True innovation, which can open doors to greatly enhanced therapeutic value, arises from a transdisciplinary approach. For example, the expanding development of complex combination products results from continuous technological advancements in drug and biological research, combined with those in engineering and manufacturing of drugs, biologics, and devices (1).

Combination products are therapeutic and/or diagnostic products that combine drugs and medical devices and consist of mixtures of drug/device, biologics/device, or even drug/device/biologics. These products are considered a vital part of healthcare since they represent a large and growing component of the therapeutic landscape (2). Despite having an official definition under the European Regulations, combination products are still challenging from a regulatory point of view due to the countless possible combinations that require a unique regulatory approach (case-by-case). Since the concept of “biologics” varies worldwide, and considering that this discussion mainly concerns the European regulatory framework, which perceives drugs and products of biological origin both as medicinal products, this paper will focus solely on drug-device combination products. Nevertheless, although the decision-tree herein concerns an European application, the United States regulations will also be addressed throughout this paper to better explain the rationale that originates the decision-tree in question.

Drug-device combinations (DDC) are regulated either by Regulation (EU) 2017/745 or by Directive 2001/83/EC depending on their principal mode of action (PMOA). However, novel complex products do not have a clearly defined PMOA, which emphasizes the lack of means of systematization as a challenge to the classification of combination products.

The several disciplines used in the development of combination products have been extensively studied and reported in the literature, but the majority of publications that address this class of products appeared in the last decade. In 2008, Gopalaswamy and Gopalaswamy (3) provided the first in-depth look at the field of combination product development, exploring the technical, scientific, regulatory and quality issues that arise when combining drugs, biologics and devices into a single product. In 2012, Couto et al. (4) addressed drug-device combination products and the regulatory challenges introduced in the medical products market by their unique dynamic, in addition to presenting case studies of transdermal patches and drug-eluting stents. However, since then, several developments occurred in the combination products field. The purpose of this article is to discuss more recent developments in the area of drug-device combinations that reflect new technological and scientific progress, exploring both the US and European regulatory framework based on the U.S. Food and Drugs Administration (FDA) and European Medicines Agency (EMA) publications on the topic, respectively.

The interest and expectations for the medical technology sector are presented, for example, in MedTech's “Innovation in Medical Technologies – Reflection Paper” (5). Over the last decade, the number of European patent filings in the MedTech field doubled, while pharma applications were relatively stagnant. According to the European Patent Office's statistics (6), in 2020 there were 8,589 patent applications in the field of pharmaceuticals. These numbers easily contrast with the 14,295 patent applications to the MedTech fields. The medical technology sector is undoubtedly growing, and it is expected that an increasing number of innovative products will reach the market. Moreover, the EMA's “Strategic Reflection” to 2025 (7) sets numerous ambitious goals, including the creation of an “integrated evaluation pathway for the assessment of medical devices, *in vitro* diagnostics, and borderline products,” with the latter being a designation that can include combination products. Therefore, it is reasonable to assume that the convergence of sectors that generates combination products will continue.

Patient-centered therapeutics has progressed immensely within healthcare, leading to increased interest in personalized care. A relevant example is custom-made medical device, which can be manufactured by three-dimensional (3D) printing processes (8). Also known as additive manufacturing, 3D printing has motivated the manufacture of custom-made products that expanded the so-called personalized medicine. 3D-printed devices have been increasingly demanded, due to their faster and more affordable manufacturing process, which results in highly customized products (9) designed to fit each patient's needs. In fact, another goal of EMA's Strategic Reflection consists of facilitating the “implementation of novel manufacturing technologies,” such as 3D-printing processes (7).

Such innovative trends demand a modern, flexible regulatory approach to deal with the inevitable complexity of the resulting products, and thus ensure their quality, safety and performance (10). To that end, regulators should be able to predict innovation and anticipate these combinations, to be prepared to assess them in the future. In this context, the main aims of this work are the critical analysis of the present regulatory framework on combination products, specifically 3D-printed scaffolds and the proposal of a decision tree for custom-made medical devices with incorporated medical substances, taking into account the risk management and the clinical and safety evaluation.

## Materials and methods

Data was collected through computational search engines, regulatory agencies and equally relevant associations, such as MedTech Europe. This data was analyzed and allowed a state-of-the-art review, a description of the decision-making process performed in the European regulatory framework, and two case studies that culminated in the proposal of a decision-tree scheme. The case studies focus on a combination with an unclear PMOA: custom-made 3D-printed bone scaffolds with incorporated medicinal substances whose function changes depending on an external stimulus.

## Findings

### Background/context

**Figure 1:**
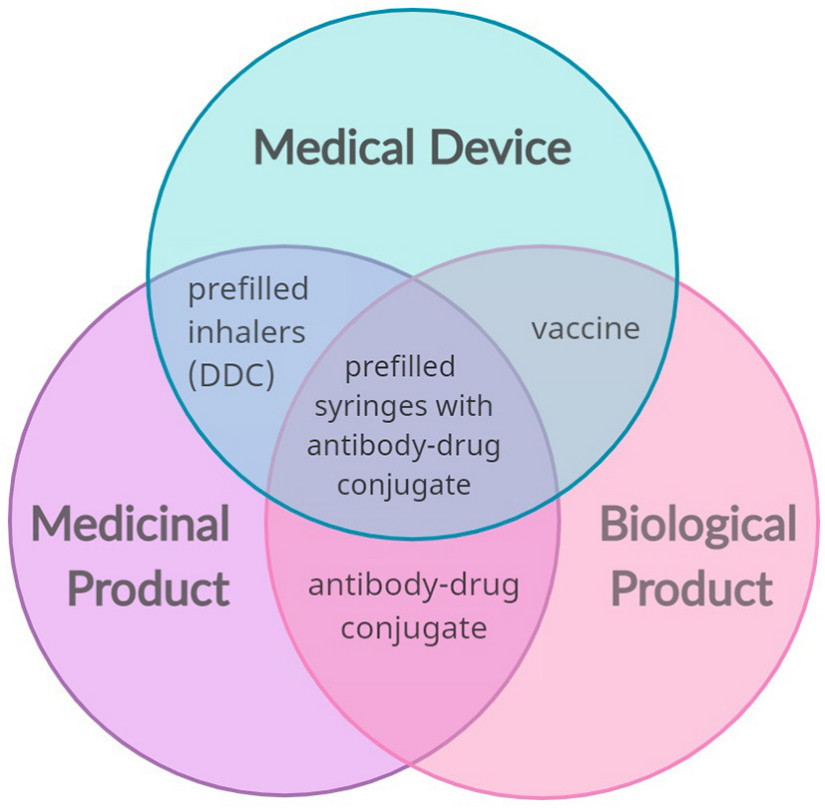
Possible convergence of the main sector of medical products (medical devices, medicinal and biological products) that results in combination products (examples: prefilled inhalers, vaccines, antibody-drug conjugates, and prefilled syringes with antibody-drug conjugates). The combination of a medical device and a medicinal product creates a drug-device combination (DDC).

Combination products are innovative and cutting-edge healthcare products. These drugs and/or biologics, device combinations have the potential to enhance the safety, effectiveness, tolerability, and patient adherence to a certain treatment, through their controlled drug release, targeted drug delivery and ease of use. In many cases, these products are developed to improve the function of clinically approved products, but it is the incorporation of novel technologies that holds great promise for advancing patient care (11, 12). Combination products have a distinct set of both benefits and risks compared to other products for similar uses and the more complex the product is, the more difficult it is to evaluate these risks and benefits properly (10). This paper focuses on drug-device combinations, but several other products fall within the scope of “combination products,” as summarized in Figure 1.

Combination products are distinct from borderline products. Borderline products are complex healthcare products for which there is uncertainty over which regulatory framework to apply, and include not only medicinal products and medical devices, but also cosmetics, biocidal products, herbal medicines and food supplements. Thus, a combination product may constitute a borderline case, but borderline products are not always combination products. Furthermore, combination products differ based on the type of packaging, being classified into single entity or integral combination products, and medicinal products with co-packaged or referenced devices. The first type occurs when the components form a single integrated product (e.g., patches for transdermal drug delivery), while the latter consists of separate items, which may or may not be contained in the same pack (e.g., reusable pen for insulin cartridges) (12).

In recent years, it has been increasingly difficult to classify combination products due to the increasing complexity of these products that is rapidly expanding as science and technology evolve at an unprecedented rate (10, 13). What started with a few simple combinations such as prefilled drug syringes or common inhalers has now evolved to a category that includes bone cements with antibiotics, transdermal systems, sensors embedded in tablets, among others.

### Regulation and the principal mode of action

The regulations for combination products are rather recent, only available on certain markets, and thus there are no specific regulatory submission formats. Consequently, a combination product is submitted either as a drug or as a device, and the whole process depends on the PMOA. The PMOA is usually defined as the therapeutic action that is expected to make the greatest contribution to the overall intended therapeutic effect of the combination product. For most products the PMOA is predictable (14) but for less common, more complex combinations, the purpose may not be so clearly defined.

The European Union (EU) deals with combination and borderline products by separating the different components, which are governed by different documents. The EU follows a complementary approach, where the combination product is not governed in full by one regulation or the other, and the determination of the PMOA plays a key role by helping decide which document will be mainly applied, without excluding other relevant requirements. To guarantee the safe entry of the product into the market, the application of a regulation or directive is complemented by the specific requirements of other relevant legislation.

In the USA, the regulatory approach to combination products is different. Regulation 21 Part 3 from *Code of Federal Regulations, Product Jurisdiction*^1^, contains most of the United States regulations. It includes the definitions for a combination product and the procedures for the FDA to determine which primary agency will provide premarket review and post-market control of a product. This code constitutes the basis of regulations, but it is the FDA's “Federal Food, Drug and Cosmetic Act” that holds the responsibility for combination products (2). The FDA is a fundamental regulatory pillar in the USA. To deal with combination products, the Office of Combination Products assigns the primary agency center to regulate the products that fall within the scope and that will determine the PMOA. Combination products might be reviewed by the following FDA centers: the Center for Biologics Evaluation and Research, the Center for Drug Evaluation and Research, and the Center for Devices and Radiological Health. Regardless of which center gets primary jurisdiction, all three must be aligned to assure an effective interaction, to achieve regulatory approval and safe introduction of products on the market (2, 13, 15).

The main difference between the EU and USA regulatory frameworks is that while the FDA deals with drug-device combination products as a whole, managed by a specifically dedicated office, in the EU the different components are separated so distinctive regulations can be applied.

#### European Union – The medicinal products directive and the medical device regulation

The pathway for approval of a drug-device combination product requires the intervention of a Notified Body (organization designated by an EU country to assess the conformity of certain products before being placed on the market), which deals with the device constituent parts, and an authority primarily responsible for the authorization of medicinal products^2^. Depending on the nature of the substances, which determines what procedure to be applied, this authority might be a Competent Authority, or the EMA.

Combination products are known for their variety and subsequent diversity of regulations, therefore, a “one-size-fits-all” approach to data requirement is not possible. Regarding drug-device combinations, there are two possible regulatory pathways to approval, depending on which medicinal component, medicinal or device, is priority hence establishing the main regulatory framework to be applied.

Medicinal products for human use are governed by Directive 2001/83 /EC, which handles all combinations that mainly have pharmacological, immunological, and metabolic actions. This Directive regulates all the medicinal products for human use, which are numerous, thus it is mainly known as the Medicinal Products Directive. All requirements for the production, classification, distribution, labeling, sale and advertising of medicinal products are established by this directive, including the procedures for marketing authorization. This includes, for example, the requirements for the technical dossier of the product. The dossier is organized into five modules, the first focusing on the administrative and prescribing information, the second including all technical summaries, module 3 addressing quality issues, while modules 4 and 5 contain non-clinical and clinical study reports, respectively. Although Directive 2001/83/EC does not provide further details concerning drug-device combinations, it does explore the meaning of an “integral part,” stipulating that the device must be combined with the drugs at the time of manufacture, application or administration of the finished products. In such case, the information related to the medical device—for example, the choice and intended function of the device and demonstration of compatibility with other components of the product, is crucial for its evaluation. The Directive also introduces the concept of a well-established medicinal use. This is useful when dealing with a medical device that incorporates well-known substances with well-established applications. A well-known medicinal substance has a different dossier for its marketing authorization application—modules 1, 2, and 3 are identical to those of other applications, while modules 4 and 5 might be covered by a detailed scientific bibliography. Also, the Notified Body that assesses the device constituent part can seek an opinion on the drug component either from a national Competent Authority or from the EMA, whenever the Agency has already evaluated a medicine containing the same substance. Therefore, in cases of combinations that include a well-known substance, the original safety data of the medicine may not be required but rather addressed by literature sources, experience, or other reliable information available.

Regulation (EU) 2017/745, also known as the new Medical Device Regulation (MDR), deals with combinations with a physical PMOA. The definition of a combination product, according to this regulation, consists of a device that incorporates a substance as an integral part. This substance must meet three requirements: (1) if used separately, it is considered a medicinal product within the meaning of Article 1 of the Medicinal Products Directive (MPD); (2) it must be liable to act upon the body; and (3) its action must be ancillary. Regarding the medical device component part, its assessments are under the responsibility of the trio manufacturer, Notified Body, and Competent Authority, but only the Notified Body approves the device. The notified bodies determine if a product meets the applicable requirements for *Conformité Européenne* (CE) marking, providing pre-marketing conformity assessments. The marketing authorization application shall include a CE certificate or an opinion from a Notified Body on the conformity of the device. This constitutes the evidence needed on the conformity of the device part with the relevant general safety and performance requirements. Once the product is allowed to display the CE mark, showing that the device conforms with European norms, it is one step closer to entering the market.

Moreover, this regulation distinguishes two types of integral products. The first type consists of devices that incorporate medicinal substances as an integral part, whose regulatory framework is laid down in Article 1(8). When the action of the incorporated medicinal substance is primary, the combination is governed by Directive 2001/83/EC, complemented with relevant safety and performance requirements of the Annex I of the MDR. On the other hand, when the action of the medicinal substance is ancillary to the action of the device, the product is then regulated as a medical device, meaning it must be CE marked. As for the second type—medical devices intended to administer medicinal products, Article 1(9) establishes the correct procedure. If the medicinal product and administration device are (1) marketed as a single integral product, (2) intended exclusively for use in the given combination, and (3) not reusable, the product is mainly regulated by the Directive 2001/83/EC. Despite this scenario not directly addressing the concept of PMOA, the designation of these products allows inferring if the action of the device is ancillary. In all other cases where these three requirements are not fulfilled, the medical device framework governs the administration device.

**Figure 2:**
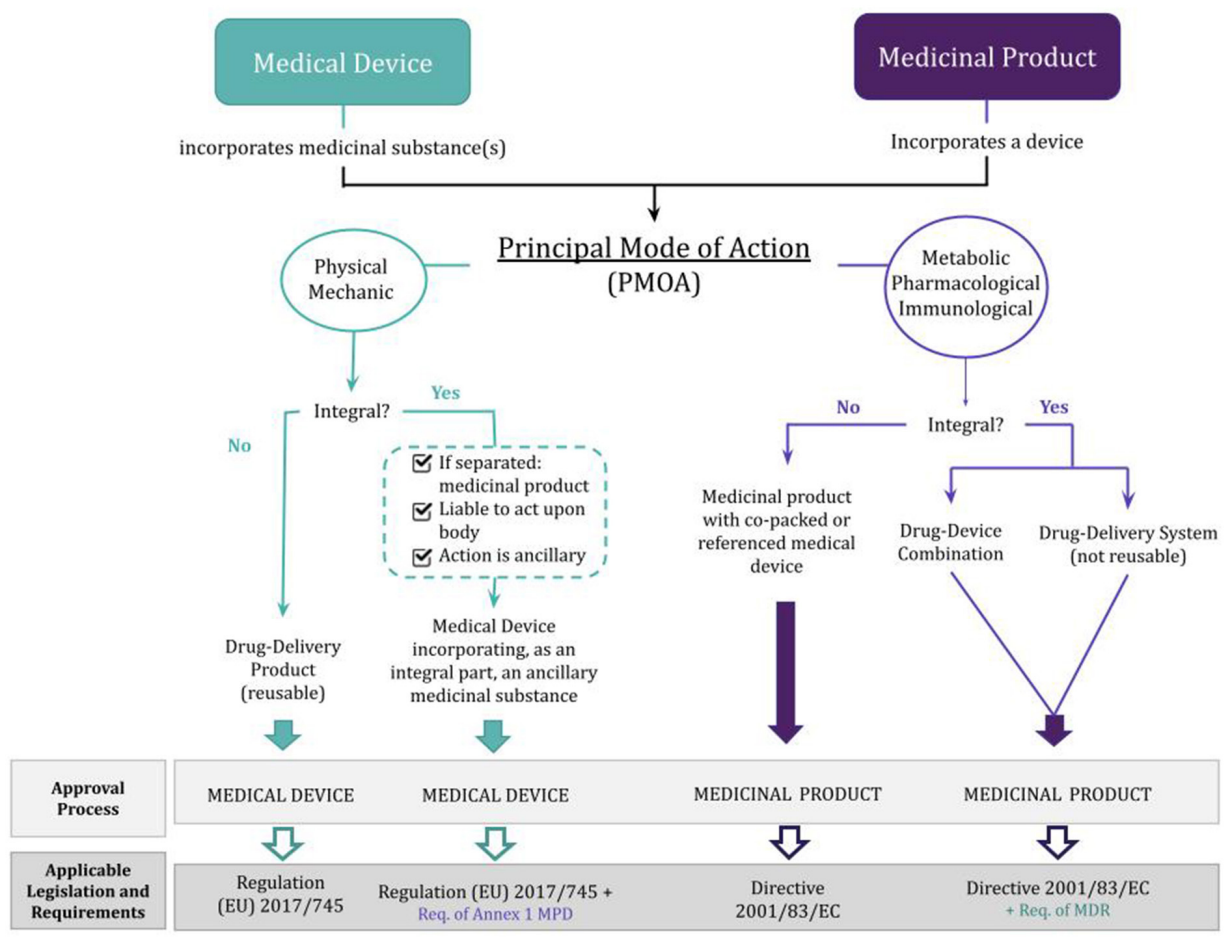
Classification and regulation of drug-device combination products depending on whether the principal mode of action is that of a medicinal product or a medical device. MPD, Medicinal Products Directive (Directive 2001/83/EC); MDR, Medical Device Regulation [Regulation (EU) 2017/745]; Req., requirements.

To better understand these different pathways for approving a combination product, Figure 2 summarizes the above-mentioned information.

#### Auxiliary guidance

Because guidelines for combination products is so complex, some non-legally binding guidelines are considered helpful for manufacturers that are unfamiliar with drug-device combinations, namely the 2021 “Guideline on the quality documentation for medicinal products when used with a medical device^3^,” from the EMA, and the MEDDEV 2.1/3 rev.3 guidance document^4^.

The EMA's guideline offers guidance on the documentation expected for drug-device combinations to be included in the quality section of the dossier for the marketing authorization application. It also clarifies expectations of Directive 2001/83/EC and addresses new obligations and concerns from Regulation (EU) 2017/745, particularly Article 117. The core precept of this guideline is that the Competent Authority for the regulation of medicines evaluates the device-specific aspects of safety and performance relevant to the quality, safety, and efficacy of the medicinal product, while the Notified Body assesses the relevant general safety and performance requirements of the device. This guideline provides the basic requirements expected in a quality dossier for marketing authorization. However, if the drug-device combinations incorporate any new emerging technology, it is recommended that applicants engage with the competent authorities and notified bodies promptly. One highlighted issue concerns the impossibility of covering all types of devices and/or future technological developments that may raise questions or even require more complex scientific assessments, this being the reason for the case-by-case analysis that regulators usually apply to combination products. The main intention of this guideline is to increase the transparency and consistency of information in these regulatory submissions.

The MEDDEV guidance document provides multiple definitions and respective examples, as well as information on consultation procedures and necessary documentation. Additionally, it assists manufacturers in distinguishing physical means from pharmacological, immunological, and metabolic means, to classify their products correctly. Since it was published in 2015, this revision of the MEDDEV was meant to complement Directives 90/385/EEC and 93/42/EEC. Despite the emergence of the MDR, this is still an essential document to clarify exactly what possible combination products exist on the market.

Another useful resource is the Helsinki Procedure 2021^5^, intended to allow consultation among competent authorities on borderline and classification issues concerning medical devices, and to ensure that appropriate guidance is published in the “Manual on Borderline & Classification for Medical Devices^5^.” This resulted from the 2021 update of the system agreed at the Medical Device Competent Authorities Meeting in Helsinki in October 2002 and followed the implementation of Regulations (EU) 2017/745 and (EU) 2017/746.

In the absence of a harmonized and global definition of combination products, Table 1 gathers definitions used in EU legislation, when referring to these products.

**Table 1:** Definitions of combination products in different EU legislations.

Medicinal Products Directive	≪The medical device or the active implantable medical device may be an integral part of the active substance≫
*PART IV, 3.4.2*	
Medical Device Regulation	≪Any device which, when placed on the market or put into service, incorporates, as an integral part, a substance which, if used separately, would be considered to be a medicinal product as defined in point 2 of Article 1 of Directive 2001/83/EC, including a medicinal product derived from human blood or human plasma as defined in point 10 of Article 1 of that Directive, and that has an action ancillary to that of the device≫ Or ≪Any device which is intended to administer a medicinal product as defined in point 2 of Article 1 of Directive 2001/83/EC≫
*Article 1(8) and 1(9)*	
MEDDEV 2.1/3 rev3	≪Medicinal products, within the meaning of Article 1 of Directive 2001/83/EC incorporated, as an integral part, in a medical device and which are liable to act upon the body with action ancillary to that of the device≫ Or ≪Medicinal product constituents or medicinal products derived from human blood or human plasma, within the meaning of Article 1 of Directive 2001/83/EC, incorporated, as an integral part, in a medical device and which are liable to act upon the human body with action ancillary to that of the device≫ Or ≪Device that is intended to administer a medicinal product within the meaning of the MPD (...) governed by the MDD or by the AIMDD without prejudice to the provisions of Directive 2001/83/EC with regard to the medicinal product≫
EMA's guideline on the quality documentation for medicinal products when used with a medical device	≪Medicinal products which contain one or more medical devices(s) as an integral part of the composition, as well as medicinal products for which one or more medical device(s) and/or device component(s) are necessary for use of the medicinal product≫
EMA's recommendation on ancillary substances incorporated in medical devices	≪Medicinal products within the meaning of Article 1(2) of Directive 2001/83/EC incorporated, as an integral part in a medical device and which are liable to act upon the body with action ancillary to that of the device≫ Or ≪Medicinal product constituents or medicinal products derived from human blood or human plasma within the meaning of Article 1(10) of Directive 2001/83/EC incorporated, as an integral part in a medical device and which are liable to act upon the human body with action ancillary to that of the device≫

### Case studies

#### Custom-made device and 3D printing

According to the MDR, a custom-made device is any device specifically made in accordance with a written prescription for specific design characteristics that is intended for the sole use of a particular patient, to exclusively meet his/her individual conditions and needs. Therefore, these devices are also governed by Regulation (EU) 2017/745, Annex XIII. Custom-made devices do not follow the same requirements as regular medical devices, since they are exempt from: a Unique Device Identification (UDI) system, technical documentation, CE mark, clinical evaluation report, post-market surveillance plan, among others. Instead, they require a technical file as described in Annex XIII of the MDR, with a statement that includes the following information, necessary for the device's approval:

- Name and address of the **manufacturer**, and of all **manufacturing sites**.
- If applicable, the name and address of the **authorized representative**.
- Data allowing **identification of the device** in question.
- A statement that the device is intended for exclusive use by a particular patient or user, identified by **name, an acronym or a numerical code**.
- The name of the **person who made the prescription** and who is authorized by national law by virtue of their professional qualifications to do so, and, where applicable, the name of the health institution concerned.
- The **specific characteristics of the product** as indicated by the prescription.
- A statement that the **device in question conforms** to the general safety and performance requirements set out in Annex I and, where applicable, indicating which general safety and performance requirements have not been fully met, together with the grounds.
- Where applicable, an **indication that the device contains or incorporates a medicinal substance**, including a human blood or plasma derivative, or tissues or cells of human origin, or of animal origin as referred to in Regulation (EU) No. 722/2012.

The Medical Device Coordination Group released a guidance document addressing some questions regarding custom-made devices, in 2021. This guidance reflects an increased interest in these products and conveys how modern state-of-the-art technologies may be used in the manufacture of custom-made devices. An example of these technologies is 3D printing.

As an additive manufacturing technique, 3D printing enables customized fabrication of 3D complex and precise structures by stacking materials layer by layer based on computer aided design software or images obtained from computed tomography and magnetic resonance imaging (8, 16). With the help of these techniques and software, an increasing number of custom-made devices are being produced resourcing to 3D printing processes, which also helped expand the so-called personalized medicine (8). Personalized medicine aims at creating highly customized products designed to fit each patient's needs and the medical 3D printing market is expected to maintain significant growth due to the huge demand for custom-made medical products (17).

The main applications of 3D printing in medicine include tissue engineering models, anatomical models, pharmacological designs, medical apparatus and instruments. Medical implants fabricated by this additive manufacturing technique have better surface, mechanical properties, and biocompatibility, compared with traditional manufacturing methods. Consequently, 3D-printed implants have been applied to major medical fields such as dentofacial, tracheobronchial, cardiovascular, orthopedics, skin wound healing, amongst others (18). Thus, there is a real need for a fast standardization of regulations for these medical products (17).

3D-Printed devices fall within the scope of the MDR if they are specifically made to fit the needs of an individual patient. However, if a medical device is 3D-printed, the manufacturer must always seek its approval, and the navigation of EU regulations is not an easy task. For this reason, despite how innovative 3D printing processes can be, they are not usually physicians and surgeons' first option for treatment (19).

Another challenge of 3D printing is the importance of the used printing software, which greatly defines the quality and safety of the product. Unlike in regular manufacturing, the software is not a mere manufacturing tool, but it must assure that the use of printed personalized devices carry a minimal risk for the patients. This concern is reflected in the gradual implementation of guidelines focused on software, such as the “Guidance on Qualification and Classification of Software in Regulation (EU) 2017/745—MDR and Regulation (EU) 2017/745—IVDR.” From our point of view, if segmentation and design software are properly addressed in current legislation, healthcare professionals might become more comfortable with pursuing these innovative possibilities of treatment, since the fulfillment of all requirements from XIII of the MDR will become easier.

It has been established that additive manufacturing processes carry a high degree of customizability but, unfortunately, there is still some variability, which challenges meeting regulatory standards and quality assurance of personalized medical devices (20). Nevertheless, Willemsen et al. (19) reported a study showing that additive manufacturing can be a reality in a hospital setting, if the needed requirements are met, intending to motivate physicians to treat unique and difficult-to-treat conditions using 3D-printed instead of conventional approaches.

#### Personalized 3D-printed scaffolds

Scaffolds obtained by different 3D-printing technologies (e.g., extrusion-based, inkjet) as sparked much interest because their performance properties can be tuned according to a specific aim (18). Moreover, multifunctional scaffolds can be combined with drug delivery strategies with or without the use of external stimulus (e.g., magnetic, electric, phototermal) (21). To address these customized scaffolds a diverse set of advanced manufacturing 3D-printing approaches, biomaterials, bioactive compounds, and drugs are under evaluation and numerous *in vitro* and *in vivo* studies are continuously being published.

For example, orthopedics is one of the most advanced fields that integrates 3D printing aiming the development of personalized bone scaffolds. These scaffolds are intended to fill bone defects that can originate from infections, tumors, trauma, or surgery (22). Recently, Saraiva et al. (23) fabricated a 3D scaffold loaded with an antibiotic (minocycline) and bioactive nanoparticles [hydroxyapatite (HA) and superparamagnetic iron oxide (SPIONs)] to be used in bone space filling and infection management. Because bacteria tend to form biofilms in non-viable implanted materials leading to implant-associated infections, the aim of the work was to test if the develop scaffold combined the enhanced osteogenic stimulation of the HA and SPIONs with the antibacterial effect of minocycline. This was the first report describing the combination of these three compounds in a 3D printed structure that can potentially be used for bone treatment, while addressing the risk of bacterial infections.

While 3D-printed platforms offer a temporary framework that provides a suitable environment for cell growth that aids in bone regeneration, these SPIONs have also been investigated by others due to their potential for the treatment of bone cancer disease by magnetic hyperthermia (24). Malignant tumors of bone and soft tissue represent a heterogeneous group of neoplasms, accounting for around 1% of all cancers in European populations (25). Although in general bone cancer has an intermediate survival rate, the mortality is highly dependent on the tumor location in the body, and the possibility of using advanced multifunction treatments is promising. SPIONS have emerged as an attractive alternative for targeted delivery of drugs because of their unique magnetic characteristics (26). In this case, the application of an external magnetic field to a scaffold containing SPIONs promotes their concentration in a target location, and the change in the iron magnetization state releases heat that can trigger the delivery of anticancer drugs (e.g., doxorubicin) or directly kill cancerous cells, resulting in a more effective triggered targeted therapy (24, 26). Moreover, without the application of an external magnetic field, the SPIONs would enhance the osteogenic and osteoconductive effects of the hydroxyapatite nanoparticles favoring bone growth as demonstrated *in vitro* by using human bone marrow derived mesenchymal stromal cells (23).

The other example refers to scaffolds designed for skin wounds applications. Liu et al. (27) developed a 3D printed alginate-gelatin hydrogel scaffold for localized cancer therapy and tissue regeneration. In brief, a core/shell fiber scaffold was fabricated by coating a homogeneous layer of polycaprolactone (PCL) on the 3D printed alginate-gelatin hydrogel scaffolds. The PCL coatings could reduce the free diffusion of drugs from the core gels. Subsequently, polydopamine (PDA) was coated on the Gel/PCL core/shell scaffolds, endowing the scaffolds with great photothermal effects. Thus, near-infrared (NIR) laser triggered on-demand drug release was realized in this system due to the thermally induced sol-gel transition of the core gels. The authors showed that the released drug (doxorubicin) and photothermal therapy could effectively prohibit or ablate tumor *in vitro* and *in vivo*. Additionally, without the application of the external stimulus the Gel/PCL/PDA core/shell scaffold could serve as platform for promoting wound healing thanks to the improved hydrophilic surface of PDA coating.

#### Innovative 3D-printed combination products: Drug or device?

The previous two examples explore 3D-printed personalized bone (23) or skin (27) scaffolds with the possibility of incorporating medicinal substances: a tetracycline antibiotic (minocycline), nanohydroxyapatite and SPIONs in the case of the bone scaffold and doxorubicin and polydopamine with respect to the skin scaffold.

Looking at the bone scaffold when no external magnetic field is present, the PMOA of the product is physical (filler and scaffold) to facilitate bone formation, with the product being classified as a medical device. In this case, the bioactive nanoparticles hydroxyapatite and SPIONs promote osteogenesis while the antibiotic minocycline prevents infection, meaning its action is also ancillary. If an external magnetic field is applied, the main clinical goal is the treatment of bone cancer by magnetic hyperthermia, and the case becomes challenging. Thus, this particular combination product has two possible distinct modes of action depending on a specific trigger. The problem here lies in the fact that the PMOA is unclear. If there is one mechanism of action pre-stimuli (device) and another post-stimulus (drug), which one should be used to classify the combination product and determine the product's regulation?

In the case of the skin scaffold, it has the ability of on-demand drug release triggered by external stimuli [near-infrared (NIR)] laser irradiation. The released drugs and NIR-induced photothermal effect could serve as chemo-photothermal synergistic cancer therapy and the product classified as medicine. On the other hand, without an external stimulus the scaffolds could promote wound healing apart from cancer therapy based on its surface, physical and mechanical properties, being classified as a medical device due to its PMOA.

Hereinafter, the option for these products will be pondered.

## Discussion

The complexity of the examples presented here resides not only on the combination product itself but on its regulatory requirements, which depend on whether it is classified as a device or a medicinal product. First, for this combination to be classified as a medical device, it would consist of a custom-made device. As explained previously, custom-made devices have some exemptions, and it is unlikely that those requirements are intended for products very complex as the ones previously described. The MDR does require an indication if the custom-made device contains or incorporates a medicinal substance but does not provide any further information about what to do concerning such substance. Alternatively, to consider the product a simple medicine incorporating a device would not be appropriate, since usually a Certificate of Conformity is required to market such products, which custom-made devices do not possess. Thus, in this case, the typical complementary approach of the EU would not work.

The solution we propose for these case studies is a tool for the schematization of the rationale used, ending on regulatory approaches based both on the FDA's streamlined approach to avoid overlapping and better adapt to complex situations (the FDA's “Final Rule”), but also on what is already the standard procedure in the EU. That solution consists of a merged regulation approach that would be an adjustment to the EU complementary approach, to properly cover the combination product. Although this is not a simple solution, the developed products are not simple either.

The choice of such a merged regulation would still ultimately result in the regular options of typical combination products—the classification as a medical device or as a medicinal product. Nevertheless, to reach a classification of an innovative combination product, several aspects would have to be considered, such as, for example:

– the medicinal substance in question (whether it is well-known or not) and its absorption;
– the intended action of the product as a whole;
– the urgency of the patient's situation;
– the duration of the different mechanisms of action;
– the risk associated with each component;
– the manufacturing process (in this case, whether it is through 3D printing);
– the peculiarities of the trigger response (in this case, for example, the strength of the magnetic field necessary to trigger the drug delivery and its impact on the body would be analyzed).

**Figure 3:**
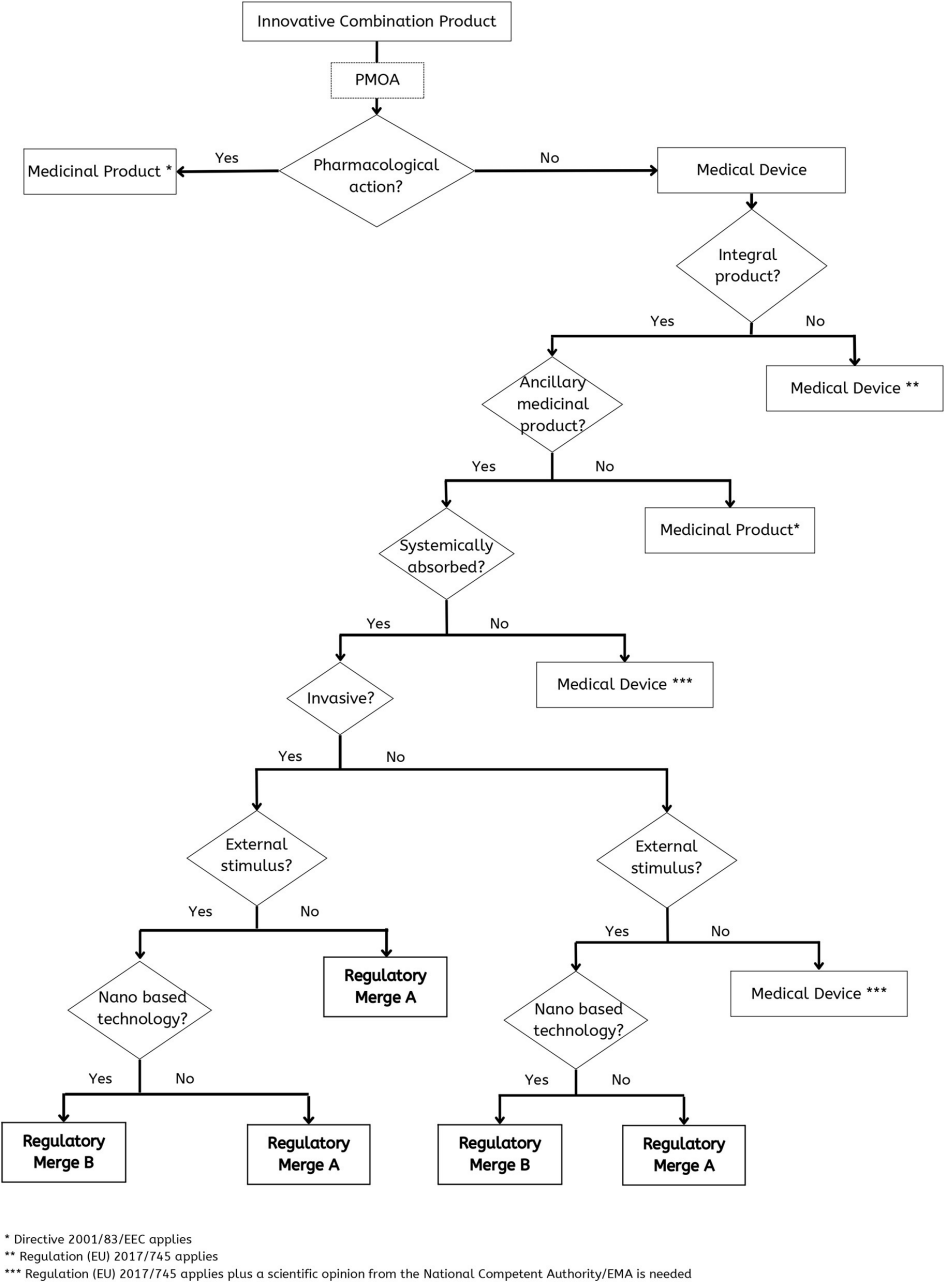
Decision-tree to deal appropriately with a combination product like a custom-made 3D-printed scaffold with medicinal substances.

However, all these aspects consist of too many variables that would be difficult for a manufacturer to assess alone. In this sense, the proposed decision-tree (Figure 3) summarizes the main aspects to assess in products of such complexity to reach a conclusion in terms of classification and consequent regulatory action.

In the case that PMOA is pharmacological there is no doubt that it is a medicinal product. In the case that the PMOA is not pharmacological the final decision is more complex, and it will depend on: (a) if it is an integral product; (b) if there is an ancillary product; (c) if it is systemically absorbed; (d) if the device is invasive; (e) if there is the need of an external stimulus and (f) if it is a nano based technology (Figure 3).

Our suggestion is to have the possibility to consider Merged regulatory approaches as will be next discussed.

### Merged regulatory approach A

This regulatory approach is the option for a custom-made medical device incorporating ancillary medicinal substances, based on Regulation (EU) 2017/745. One problem with the current pathway for custom-made devices is that regulations provide little information when the device includes a medicinal substance. Additionally, a 3D-printed scaffold does not possess the same risks as orthotic braces or hearing aids, and so it should not have identical requirements.

Taking into consideration the exemptions of custom-made devices, Merge A requires some clinical evidence that the device is safe and can achieve clinical benefits. Furthermore, the complexity of the product demands the existence of a post-market surveillance plan. Additionally, incidents and their corrective actions, undesirable effects, and feedback and complaints from the users, should also be a priority. Also, as previously highlighted by Willemsen et al. (19), the development process of each custom-made device would be facilitated if the physicians had access to details of previous similar situations and apply such knowledge to their products. Each custom-made device is unique. Nevertheless, the slightest bit of information available might be crucial to help future patients in similar situations.

Concerning the medicinal substance, Merge A would require more than the mere indication of its presence within the combination. As standard procedure, the information relevant to the medicinal substance should include the quantitative composition, details of the manufacturing method, control of critical excipients, control of the intermediate and finished product, stability, and, when appropriate, validation data. Moreover, if the substance is well known and its use is well established, as is the case of the antibiotic minocycline and the doxorubicin, the Notified Body may request an opinion from EMA or the national competent authority that previously evaluated the substance. Therefore, the original safety data may not be required but rather addressed by literature sources, experience, or other reliable available information.

### Merged regulatory approach B

This regulatory approach is the option for a medicinal substance combined with a custom-made medical device. The foundation of Merge B is Directive 2001/83/EC, meaning the product would be regulated mainly as a medicinal product, but the requirements from Regulation (EU) 2017/745 are slightly different. While there is no need for evidence of conformity, like a CE mark of conformity, there should be documentation with the manufacturing site(s) that allow the understanding of the design, manufacture, and performance of the device. In addition to regular post-market vigilance, the manufacturer should be responsible for monitoring and follow-up the patient regarding the medical device.

This would be the case when hydroxyapatite and superparamagnetic iron oxide nanoparticles and the polydopamine are present, due to their nano properties. Nanomedicines have demonstrated significant therapeutic advantages for a multitude of applications, but their translation into clinical practice has not progressed as fast as the many published positive results suggest. This is due to the current need for a strong fit-for-purpose regulatory framework for nanotechnology and its clinical applications. Thus, the nanoparticles and the nanomaterial would require further data when compared to minocycline, since they have not been as extensively studied. There might not be any major safety concerns, but these nanoparticles and materials might need stronger efficacy and safety data to support a positive risk-benefit balance.

### Decision-tree

As previously mentioned, not all innovative combination products will possess the same peculiarities and triggers and the cases presented above, and they might not even depend on nanoparticles and nanomaterials. Therefore, a decision-tree could never focus on such specific aspects or it would not be of much use to a manufacturer. In this sense, the proposed decision-tree (Figure 3) displays the pathway to reach different possibilities of regulation, namely:

– Medicinal product to which the Directive 2001/83/EEC applies.
– Custom-made medical device to which the Regulation (EU) 2017/745 applies.
– Custom-made medical device incorporating an ancillary medicinal substance to which the Regulation (EU) 2017/745 applies, but that will also require a scientific opinion from a National Competent Authority or EMA.
– Custom-made medical device incorporating an ancillary medicinal substance that requires Merged regulatory approach A.
– Medicinal substance combined with a custom-made medical device that requires merged regulatory approach B.

## Conclusion

3D Printing aims to revolutionize healthcare in the current era of personalized medicine. The advantages displayed in the described case studies are undeniable, but there is still work to do so patients can access this technology more easily. The current requirements for custom-made devices established by Regulation (EU) 2017/745 cannot fully and safely govern 3D-printed devices. This advanced technology is producing more complex devices than what was probably envisioned and expected for 3D printing. In these cases, the scaffolds not only would incorporate medicinal products, but these would be delivered innovatively. For that reason, a mixture of Regulation (EU) 2017/745 and Directive 2001/83/EC could ideally cover all the requirements to assure the scaffold's efficacy and safety. However, that mixture must be adequate according to the products' specific characteristics. Additionally, the manufacture of a personalized 3D-printed product does not depend on just one physician, but rather a multidisciplinary team which would necessarily depend on collaboration and communication. This case also highlighted 3D printing's concerns regarding the software. Nevertheless, despite the obvious aspects in need to be addressed to ease the development of such innovative approaches, and the fact that 3D printing methods in the manufacture of implantable medical devices in patients are still in their infancy, 3D printing may possibly be a recurrent tool in the near future of medicine.

This decision-tree was the result of an exercise focused on two custom-made 3D-printed scaffolds with incorporated medicinal substances. The proposed approach considers both drug and device to ensure that every aspect of the product is duly covered and presents a simple and somewhat familiar approach to the EU regulatory requirements. Thus, this tool is useful for the decision-making process applied to customized medical devices with medicinal substances, even if the device was not developed *via* 3D printing. However, this tree can only be applied if the custom-made medical devices are within the EU regulatory context, which, considering the current market of medical products, can be quite specific or even limited. Moreover, although our discussion is focused on two specific cases of 3D-printed scaffolds it can be easily perceived that the suggested decision-tree (Figure 3) can be translated to other complex products supporting the stakeholders (researchers and industry included) decision. Nevertheless, this tool for schematizing the rationale and ease the regulatory decision-making process is helpful, especially considering the continuous evolution of personalized medicine which will certainly bring to the market numerous medical products such as the one presented in this paper.

Currently, there are no regulations that can be applied to all complex combination products. The increasing complexity of these products has furthered the need to develop multidisciplinary collaborations within the health sector to understand and adapt to these innovative healthcare solutions. Considering that the examples presented in this paper involves electric field changes (23) and near-infrared laser irradiation (27), such collaborations of expertise will be vital with regards to future combination products that may offer these new technologies, so that patients can benefit from them with guaranteed quality and safety. To ensure this, communication is crucial. Regulators must follow biomedical and technological evolution, while researchers should be aware of the regulatory frameworks that apply to their work. The goal is to be prepared to support the boost development of these complex medicines that will foster and enhance personalized healthcare solutions by converging different technologies, to promote and protect human health.

## Data availability statement

The datasets presented in this study can be found in online repositories. The names of the repositories and accession number(s) can be found in the article.

## Author contributions

Conceptualization, supervision, project administration, and methodology: AB and HR. Writing-original draft preparation: MR. Writing-review and editing: MR, AB, and HR. All authors have read and agreed to the published version of the manuscript.

## Conflict of interest

The authors declare that the research was conducted in the absence of any commercial or financial relationships that could be construed as a potential conflict of interest.

## Publisher's note

All claims expressed in this article are solely those of the authors and do not necessarily represent those of their affiliated organizations, or those of the publisher, the editors and the reviewers. Any product that may be evaluated in this article, or claim that may be made by its manufacturer, is not guaranteed or endorsed by the publisher.

## Footnotes

^1^U.S. FDA. Available on: https://www.accessdata.fda.gov/scripts/cdrh/cfdocs/cfcfr/cfrsearch.cfm?fr=3.2.

^2^European Commission. Available on: https://eur-lex.europa.eu/homepage.html.

^3^EMA. Available on: https://www.ema.europa.eu/en/quality-documentation-medicinal-products-when-used-medical-device.

^4^European Commission. Available on: https://ec.europa.eu/health/index_en.

^5^Helsinki Procedure for borderline and classification under MDR & IVDR Available on: https://health.ec.europa.eu/medical-devices-sector/new-regulations/guidance-mdcg-endorsed-documents-and-other-guidance_en.
